# Editorial: The immune microenvironment-microbiome interactions in peri-implantitis and periodontitis

**DOI:** 10.3389/fcimb.2026.1844086

**Published:** 2026-06-30

**Authors:** Lucinda J. Bessa, Ying An, Zejian Li

**Affiliations:** 1 Egas Moniz Center for Interdisciplinary Research (CiiEM), Egas Moniz School of Health & Science, Almada, Portugal; 2 Department of Periodontology, School of Stomatology, The Fourth Military Medical University, Xi’an, China; 3 Hospital of Stomatology, The First Affiliated Hospital of Jinan University, Guangzhou, China

**Keywords:** immune microenvironment, microbiome, oral microorganisms, peri-implantitis, periodontitis

## Introduction

Peri-implantitis and periodontitis are highly prevalent chronic inflammatory diseases and leading causes of tooth and implant loss worldwide (Caton et al., 2018; Fu et al., 2025). They do not result solely from pathogenic microorganisms but from a dysregulated host response to a dysbiotic microbiota (Cui et al., 2025; Mehrnia and Van Dyke, 2026). The interplay between microbial communities and the local immune microenvironment governs disease progression. This disruption establishes a pro-inflammatory state that sustains immune activation, generating a self-amplifying feedback loop that drives persistent inflammation and progressive tissue destruction.

This Research Topic — *The immune microenvironment-microbiome interactions in peri-implantitis and periodontitis* — explores these complex interplays across multiple dimensions. The seven contributing manuscripts provide diverse perspectives, ranging from retrospective clinical evaluations of immediate implant placement and orthodontic appliance impact to high-throughput proteomic and metagenomic analyses of oral fluids. Additionally, comprehensive reviews in this collection detail the cellular and molecular mechanisms—such as macrophage metabolic reprogramming and neuro-immune crosstalk—that drive the persistent tissue destruction characteristic of these diseases.

## Overview of contributing articles

In a retrospective clinical study, Li et al. evaluated the short-term success of immediate implant placement in 95 patients with varying severities of periodontitis. The study reported a high overall 1-year survival rate of 97.86%, demonstrating that this approach is feasible even without prior systematic periodontal treatment. However, the researchers noted that survival rates were significantly lower in patients with Stage IV and Grade C periodontitis, suggesting that increased disease severity and rapid progression remain critical risk factors that necessitate meticulous surgical planning and comprehensive postoperative care.

The work by Blanco-Pintos et al. utilized a high-throughput SWATH-MS proteomic technique to characterize the periodontal proteome in both gingival crevicular fluid (GCF) and saliva. By comparing healthy individuals to those with Stage III-IV periodontitis, the authors identified distinct proteome structures in both oral fluids, with 25.2% of GCF proteins and 15.7% of salivary proteins being differentially expressed. Their findings highlight that protein expression patterns vary both qualitatively and quantitatively between these fluids, underscoring the potential need for fluid-specific molecular biomarkers in clinical diagnostics.

Focusing on cellular mechanisms, Jia et al. provided a comprehensive review of how mitochondrial dysfunction in macrophages serves as a central driver of periodontal pathogenesis. The authors detailed how dysregulated processes, including metabolic reprogramming, excessive reactive oxygen species (ROS) release, and impaired mitophagy, promote M1 macrophage polarization and exacerbate osteoclast differentiation. Furthermore, the review highlights emerging therapeutic strategies targeting these mitochondrial pathways, such as the use of antioxidants and nanomaterials, to restore periodontal immune homeostasis.

Investigating the influence of the local microenvironment, Yuan et al. explored how physiologically relevant hypoxia (2% O_2_) modulates macrophage behavior and its interaction with oral bacteria. They found that low oxygen tension activates the HIF-1α signaling pathway, inducing a metabolic shift toward glycolysis and a transition to an amoeboid migration mode. Despite these changes, hypoxic macrophages maintained their phagocytic capacity against both commensal and pathogenic bacteria, though they exhibited altered cytokine profiles characterized by increased IL-1β and decreased TNF-α.

The impact of orthodontic interventions on the oral environment was examined by Xu et al., who compared the effects of clear aligners (CA) and fixed appliances (FA) on periodontal health and microbial homeostasis. Their prospective study revealed that FA were associated with worse periodontal indices, enrichment of pathogenic genera like *Prevotella* and *Veillonella*, and elevated oxidative stress markers (8-OHdG) in the GCF. In contrast, CA demonstrated a greater capacity for preserving health-associated microbial taxa and minimizing oxidative stress-related damage during treatment.

Røsland et al. explored the “oral-lung axis” through a longitudinal trial assessing the effect of full-mouth periodontal disinfection on the subgingival microbiome and lung function in never-smokers. Utilizing shotgun metagenomics, the study observed a significant shift from periodontitis-associated taxa toward health-associated species, such as *Actinomyces oris* and *Rothia dentocariosa*, following periodontal treatment. Crucially, these microbial changes were associated with reductions in airway resistance, suggesting that periodontal treatment may provide respiratory benefits even in individuals without prior lung disease.

Finally, the review by Dong et al. synthesized evidence on the multi-dimensional role of Advanced Glycation End Products (AGEs) in fueling periodontal destruction. The authors detailed how AGEs directly modify the periodontal extracellular matrix (ECM) to increase tissue brittleness while simultaneously triggering self-perpetuating inflammatory cycles through the RAGE receptor. A key highlight of this work is the exploration of the neuro-immune axis, where AGEs activate sensory neurons to amplify local inflammation and contribute to clinical symptoms like pain and chronicity.

## Discussion and future directions

The evidence presented highlights that microbial composition alone is insufficient to explain disease outcomes; rather, host factors such as immune cell metabolism, oxygen tension, and systemic modifiers (e.g., AGEs) critically shape disease trajectories. Importantly, several contributions underscore that similar clinical phenotypes may arise from distinct molecular and ecological pathways, emphasizing the heterogeneity of these diseases. This variability helps explain differences in treatment responses and supports a move away from one-size-fits-all therapeutic approaches. Moreover, the identification of fluid-specific proteomic signatures and the systemic links revealed through the oral–lung axis further expand the clinical relevance of periodontal research beyond the oral cavity.

Future research should prioritize integrative, multi-omics approaches combined with longitudinal clinical data to unravel causal relationships and identify robust, predictive biomarkers. A key direction lies in translating mechanistic insights—such as macrophage metabolic reprogramming and hypoxia-driven immune modulation—into targeted therapies capable of restoring immune homeostasis rather than merely reducing microbial load. Precision medicine strategies, including patient stratification based on immune and microbial profiles, will likely be essential to improve clinical outcomes. Additionally, the development of interventions that modulate the host response, such as mitochondria-targeted therapies, redox modulation, and neuro-immune regulation, represents a promising frontier. Finally, greater attention should be given to systemic interconnections, including the oral–lung and oral–metabolic axes, to fully elucidate the broader health implications of periodontal diseases and to position periodontal therapy as part of comprehensive patient care.
